# Extracellular Mycobacterial DnaK Polarizes Macrophages to the M2-Like Phenotype

**DOI:** 10.1371/journal.pone.0113441

**Published:** 2014-11-24

**Authors:** Rafael L. Lopes, Thiago J. Borges, Jessica F. Araújo, Nathana G. Pinho, Letícia S. Bergamin, Ana Maria O. Battastini, Stéfanie P. Muraro, Ana Paula D. Souza, Rafael F. Zanin, Cristina Bonorino

**Affiliations:** 1 Laboratory of Cellular and Molecular Immunology, Biomedical Research Institute, Pontifícia Universidade Católica do Rio Grande do Sul, Porto Alegre, Rio Grande do Sul, Brazil; 2 Department of Cellular and Molecular Biology, School of Biosciences, Pontifícia Universidade Católica do Rio Grande do Sul, Porto Alegre, Rio Grande do Sul, Brazil; 3 School of Pharmacy and Laboratory of Clinical and Experimental Immunology, Biomedical Research Institute, Pontifícia Universidade Católica do Rio Grande do Sul, Porto Alegre, Rio Grande do Sul, Brazil; 4 Departamento de Bioquímica, Instituto de Ciências Básicas da Saúde, Universidade Federal do Rio Grande do Sul, Porto Alegre, Rio Grande do Sul, Brazil; Fundació Institut d'Investigació en Ciències de la Salut Germans Trias i Pujol. Universitat Autònoma de Barcelona. CIBERES, Spain

## Abstract

Macrophages are myeloid cells that play an essential role in inflammation and host defense, regulating immune responses and maintaining tissue homeostasis. Depending on the microenvironment, macrophages can polarize to two distinct phenotypes. The M1 phenotype is activated by IFN-γ and bacterial products, and displays an inflammatory profile, while M2 macrophages are activated by IL-4 and tend to be anti-inflammatory or immunosupressive. It was observed that DnaK from *Mycobacterium tuberculosis* has immunosuppressive properties, inducing a tolerogenic phenotype in dendritic cells and MDSCs, contributing to graft acceptance and tumor growth. However, its role in macrophage polarization remains to be elucidated. We asked whether DnaK was able to modulate macrophage phenotype. Murine macrophages, derived from bone marrow, or from the peritoneum, were incubated with DnaK and their phenotype compared to M1 or M2 polarized macrophages. Treatment with DnaK leads macrophages to present higher arginase I activity, IL-10 production and FIZZ1 and Ym1 expression. Furthermore, DnaK increased surface levels of CD206. Importantly, DnaK-treated macrophages were able to promote tumor growth in an allogeneic melanoma model. Our results suggest that DnaK polarizes macrophages to the M2-like phenotype and could constitute a virulence factor and is an important immunomodulator of macrophage responses.

## Introduction

Macrophages are myeloid cells which have an important role during inflammation, infection resolution, tissue repair and cancer [1]. These cells have a marked phenotypic heterogeneity, which is dependent on the microenvironment conditions. T helper 1 (Th1) or T helper 2 (Th2) cytokines stimulate macrophage to differentiate into two opposed phenotypes. Classically activated macrophages (M1) are induced by Th1 cytokines (IFN-γ), or by bacterial products (e.g LPS). They are able to control infections, have a tumoricidal activity and secrete high levels of pro-inflammatory cytokines. Alternatively activated macrophages (M2) are induced by Th2 cytokines (IL-4 and/or IL-13) and have important roles in allergy, parasitic infections and tissue repair [2]. Both phenotypes can be differentiated by surface receptors, gene expression and cytokines profile produced. M1 macrophages express CD80, CD86, produce NO and secrete the pro-inflammatory cytokines TNF-α, IL-12, IL-6 and IL-1β. M2 macrophages express CD206 and CD163. They can produce IL-10, TGF-β and show an increased arginase I activity [3]. In addition, M2 macrophage polarization can be defined based on a specific genetic signature characterized by the upregulation of Ym1 (also known as *Chil3l3*) and FIZZ1 (also known as *Retnla*) genes [4], [5].

Both in infections and tumors, a switch from Th1 (or M1) to Th2 (or M2) immunity can occur, leading to the generation of a suppressive environment that abrogates effector immunity [6]. During mycobacterial infections, the generation of suppressive macrophage populations coincides with a switch from a Th1 to a Th2 response, and such macrophages are important for the persistence of the pathogen [7]. *Mycobacterium tuberculosis* can reprogram macrophages to M2 via secretion of IL-10 [8], a major immunosuppressant that counteracts IFN-γ and TNF-α, the two major cytokines that drive the effective response that clears the infection. In tumors, macrophages infiltrate the microenvironment, and modulate T-cell and stroma activity, either promoting or inhibiting tumor progression [9]. In established tumors, macrophages are biased toward the M2-like phenotype which has tumor-promoting functions [10]–[13] correlating with poor prognosis [14], [15].

The chaperone DnaK is the major bacterial counterpart of heat shock protein 70 (HSP70) [16]. Extracellular HSP70 from different sources has been demonstrated to have protective and regulatory roles in different inflammatory disease models as arthritis [17], [18], colitis [19], transplants [20] and brain ischemia [21]. These effects were reported to be due to modulation of dendritic cells (DCs) [22]–[24] and monocytes [25] to a tolerogenic state, inducing IL-10 production and downregulating MHC class II.

Recently, DnaK was found in vesicles released by *Mycobacterium tuberculosis* [26]. Administration of these vesicles to mice before infection accelerated the pathogenesis of the disease, suggesting that it could have a role in mycobacterial infection. Nevertheless, the immune effects of prokaryotic Hsp70 in macrophages have never been addressed.

In the present study we investigated whether mycobacterial DnaK polarizes murine macrophages. Macrophages treated with DnaK behaved like M2 macrophages. Furthermore, these cells presented M2 function *in vivo* in an allogeneic murine melanoma model, enhancing tumor growth. Our results indicate that macrophages treated with DnaK become functional M2-like cells, with tumor promoting potential.

## Results

### Extracellular DnaK induces the expression of M2 markers in bone marrow-derived macrophages

**Figure 1:**
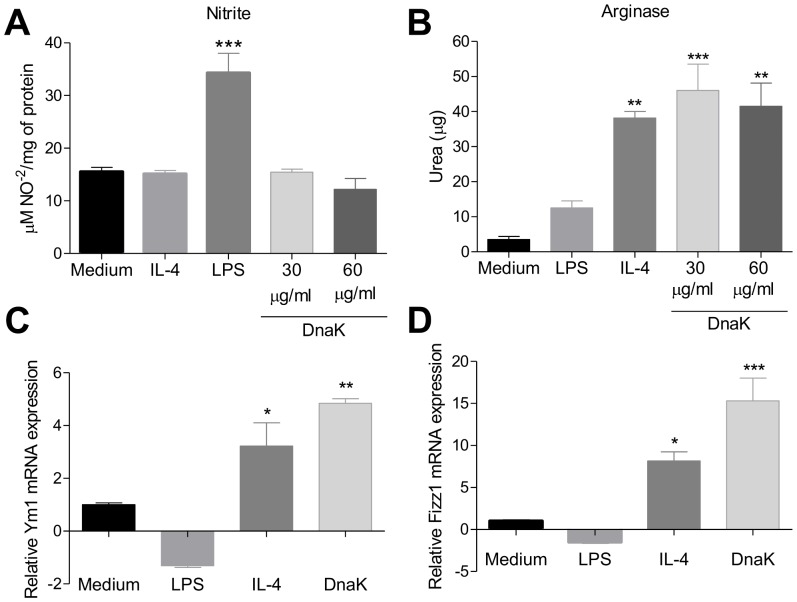
Extracellular DnaK induces the expression of M2 markers in bone marrow-derived macrophages. BMMs were treated with LPS (30 ng/ml), IL-4 (40 ng/mL), DnaK (30 µg/mL or 60 µg/mL), or left unstimulated for 24 h. (A) iNOS activity was determinated by nitrite (NO^2−^) accumulation in the supernatant of macrophages. Data are the mean ± S.D. from triplicates. Data representative of three independent experiments. (***) p<0.001 indicates difference between LPS and other treatment groups. (B) Arginase activity was assessed by measuring the formation of urea from arginine. Data are the mean ± S.D. from triplicates. (**) p<0.01 and (***) p<0.001 indicate difference between treated groups and the medium group. Effect of DnaK on Ym1 (C) and FIZZ1 (D) expression in macrophages were quantified by real time PCR. The total amount of Ym1 and FIZZ1 mRNA were normalized to β-microglobulin signals and expressed as 2^−Δ/ΔCT^. The values represent means ± SEM from triplicates. Data representative of three independent experiments. All data were analyzed by one-way ANOVA with Tukey post hoc test.

To verify the effect of DnaK treatment on macrophages polarization, we treated macrophages differentiated from bone-marrow cells (BMMs) of B6 mice with different DnaK concentrations and compared iNOS and arginase activities between cells stimulated with LPS (M1), IL-4 (M2) or untreated cells. iNOS activity was induced in M1 macrophages but not in cells treated with DnaK (30 or 60 µg/mL) or in M2 (Fig. 1A). However, DnaK treatment increased the activity of arginase in both concentrations tested when compared with control or M1 macrophages (Fig. 1B). The increase of arginase activity by DnaK was similar to the one in macrophages treated with IL-4 (Fig. 1B). Because there were no differences between both DnaK concentrations that were tested, we used 30 µg/mL in all of the following experiments.

Murine polarized macrophages exhibit a distinct gene signature which can be used as polarization-associated markers [27]. The M2 phenotype is associated with the expression of Ym1 and FIZZ1 [4], [5]. To evaluate whether DnaK can induce these M2 gene markers, we treated BMMs with LPS, IL-4 or DnaK for 24 h and then assessed both Ym1 and FIZZ1 mRNA levels by real time PCR. DnaK induced Ym1 (Fig. 1C) and FIZZ1 (Fig. 1D) mRNA expression by macrophages to levels superior to the ones in IL-4 polarized M2 macrophages. LPS treated BMMs (M1) did not express any of the two markers. Altogether, these data demonstrate that the treatment of BMMs with DnaK induces the expression of well-characterized markers associated with M2 phenotype.

### DnaK induces release of M2-like cytokines by macrophages

**Figure 2:**
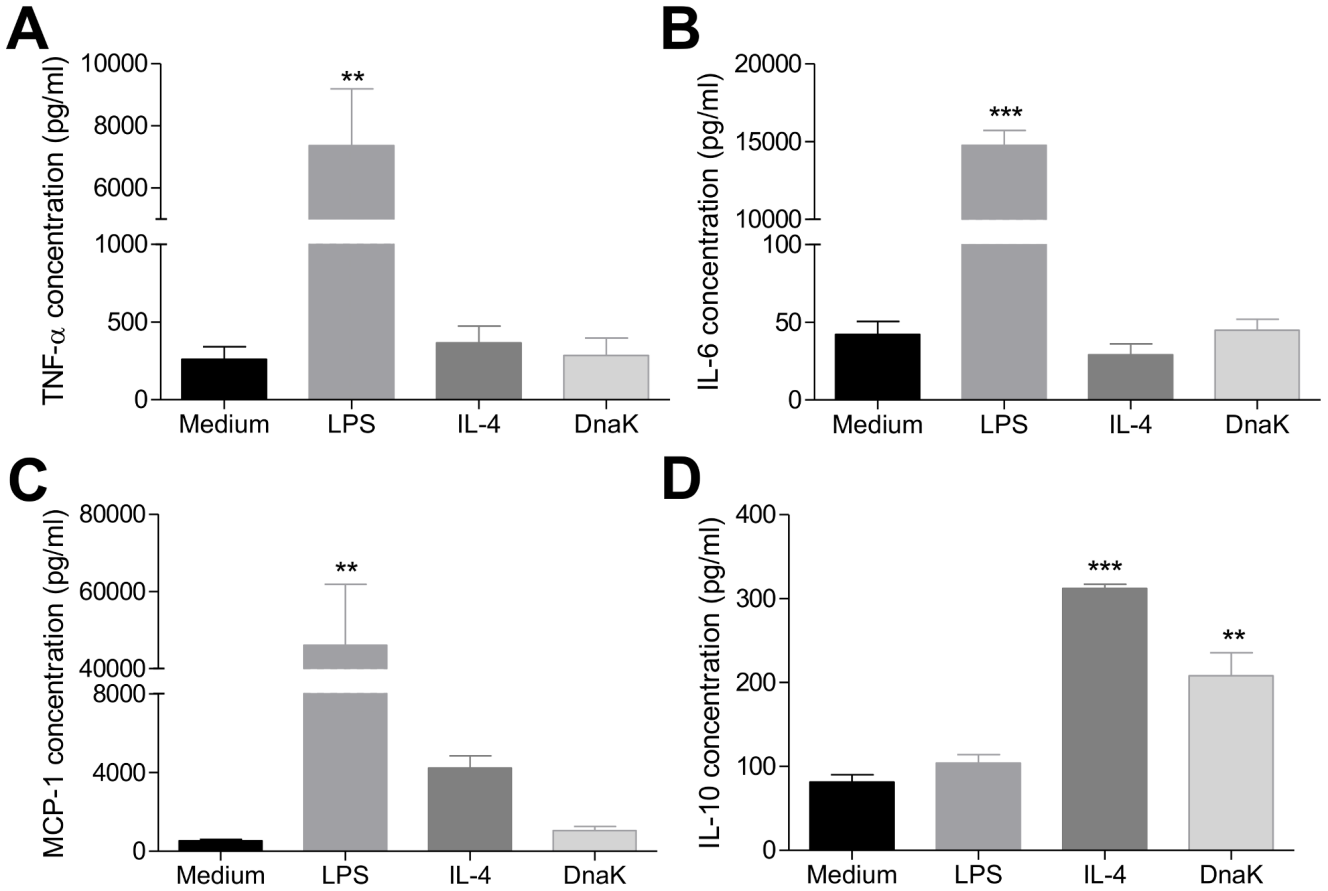
Macrophages release an M2-like cytokine profile upon stimulation with DnaK. BMMs were treated with 30 ng/mL of LPS, 40 ng/mL of IL-4, 30 µg/mL or 60 µg/mL of DnaK or left unstimulated for 24 h. (A) TNF-α, (B) IL-6, (C) MCP-1 and (D) IL-10 were measured from culture supernatants by flow cytometry. The values represent means ± SEM in pg/ml from triplicates. (**) p<0.01 and (***) p<0.001 indicate significant difference between treated groups and medium group. All data has been by one-way ANOVA with Tukey post hoc test. Data representative of four independent experiments.

To investigate the profile of cytokines released by the BMMs, we analyzed the production of TNF-α, MCP-1, IL-6, IL-10 and TGF-β upon stimulation with LPS, DnaK or IL-4 for 24 h. The treatment of BMMs with LPS led to an increased production of TNF-α when compared to control, IL-4 and DnaK treatments (Fig. 2A). Production of IL-6 was lower in the DnaK group when compared to LPS, and similar to M2 macrophages (Fig. 2B). Likewise, MCP-1 production was lower in DnaK-treated macrophages when compared to M1 and M2 phenotype and similar to the control (Fig. 2C). In contrast, DnaK or M2 macrophages produced higher levels of IL-10 when compared with LPS or control (Fig. 2D). We also evaluated the TGF-β production by treated BMMs. DnaK treatment did not induce the production of TGF-β (data not shown). Thus, murine macrophages treated with DnaK are similar to M2 macrophages, producing low levels of M1 cytokines but high levels of IL-10.

### DnaK induces M2 phenotype surface markers

**Figure 3:**
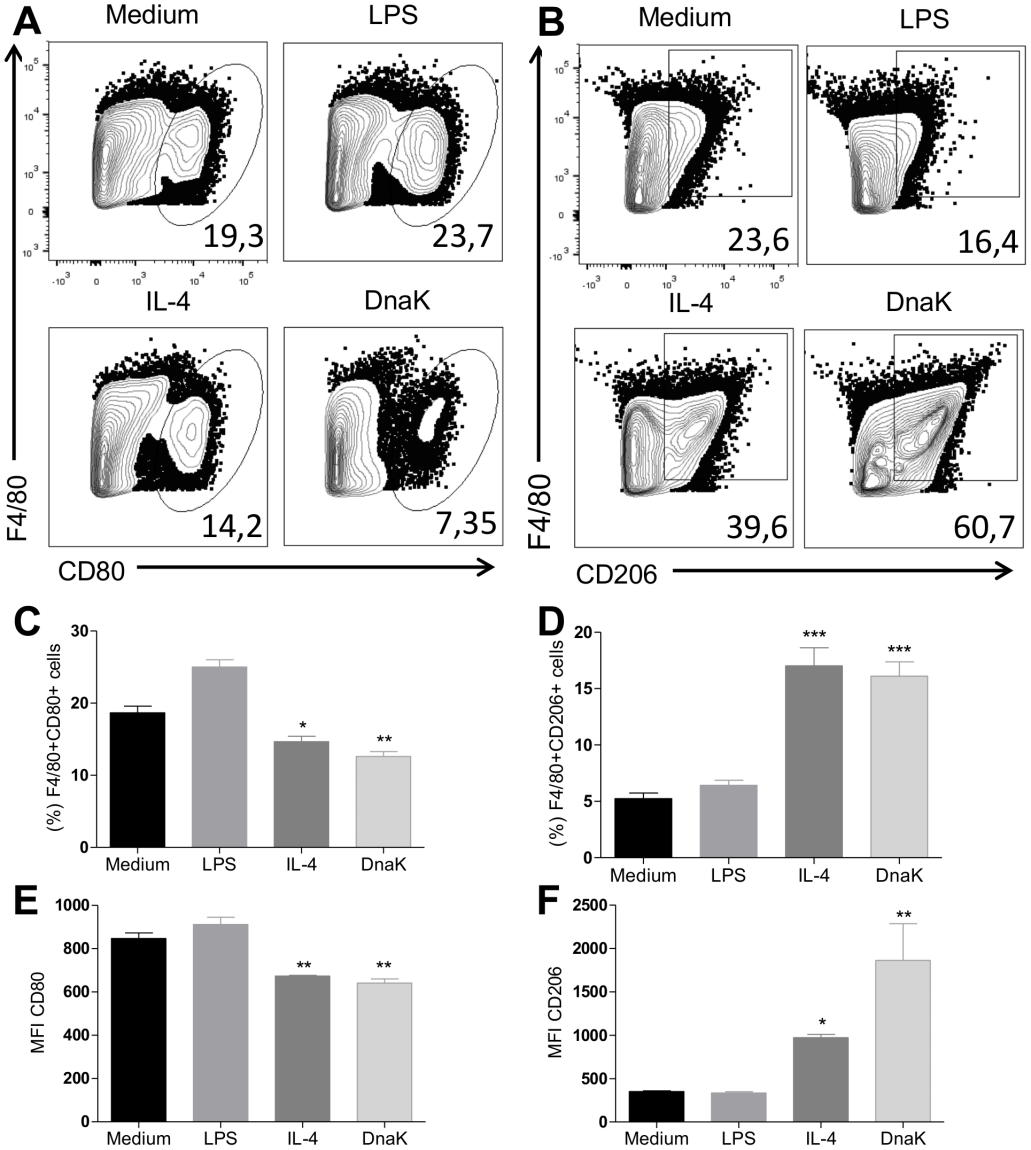
Induction of M2 surface marker CD206 by DnaK treatment. Representative dot plots of surface (A) CD80 and (B) CD206 expression in BMMs treated with 30 ng/mL of LPS, 40 ng/mL of IL-4, 30 µg/mL or 60 µg/mL of DnaK, or left unstimulated for 24 h. The percentage values of (C) F4/80^+^CD80^+^ and (D) F4/80+CD206+ cells represent means ± SEM from triplicates. (E) and (F) show respective values for MFI analyses. (*) p<0.05, (**) p<0.01 and (***) p<0.001 indicate significant difference treated groups in relation to medium group. All data were analyzed by one-way ANOVA with Tukey post hoc test. Data are representative of three independent experiments.

Classically activated macrophages (M1) express CD80 and CD86 on surface. CD206 - the mannose receptor - is a specific marker of alternatively activated macrophages (M2). To analyze the expression of these surface molecules, we stimulated BMMs with DnaK, LPS or IL-4 and analyzed them for the expression of CD206 and CD80 by flow cytometry. Figure 3A shows representative dot plots of the cell surface stainings analyzed. DnaK treatment decreased the percentage of CD80+ macrophages (Fig. 3A and C) as well as CD80 MFI (Fig. 3E) in cells. In contrast, treatment with DnaK increased the percentage of CD206+ cells (Fig. 3B and D) as well as CD206 MFI (Fig. 3F) in comparison to other treatments. These results indicated that macrophages treated with DnaK presented a profile of surface molecules consistent with that is observed in alternative activated (M2) macrophages.

**Figure 4:**
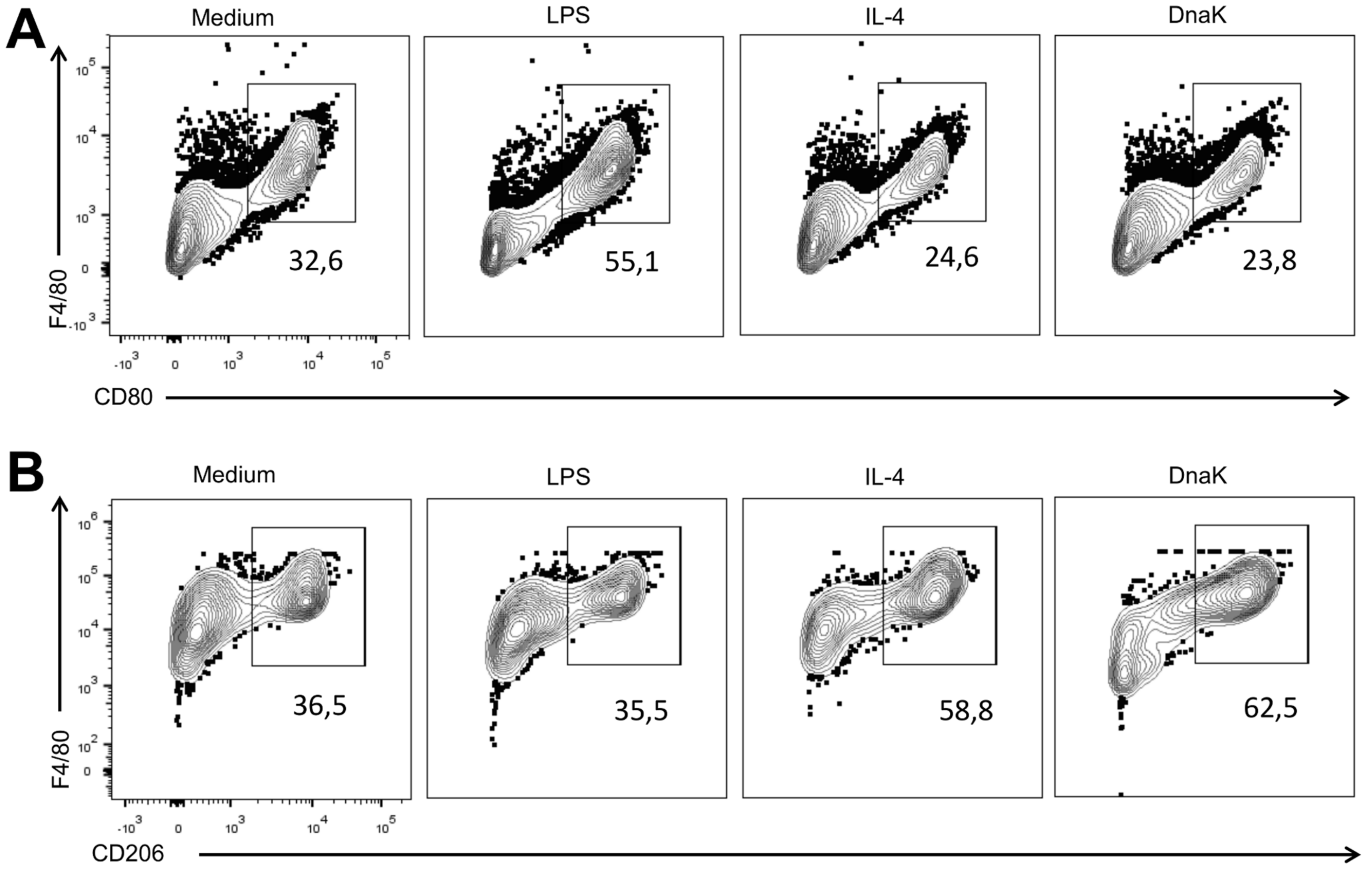
Extracellular DnaK induces the expression of CD206 in peritoneal macrophages. Peritoneal macrophages were isolated from B6 mice and treated with 30 ng/mL of LPS, 40 ng/mL of IL-4, 30 µg/mL or 60 µg/mL of DnaK, or left unstimulated for 24 h. After that, cells were analyzed by flow cytometry and data presented as representative dot plots of (A) CD80, (B) CD206 expression. Data representative of three independent experiments.

We also asked if DnaK could have modulatory effect in another macrophage population. To answer that, we treated peritoneal macrophages with DnaK and compared them with M1 and M2 macrophages. DnaK-treated peritoneal macrophages presented a lower percentage of CD80+ cells when compared control and M1 macrophages (Fig. 4A and B). Also, macrophages expressing CD80 were similar in the M2 and DnaK-treated cells (Fig. 4A and B). In contrast, macrophages treated with extracellular DnaK showed a higher expression of CD206 than other treatments, including M2 macrophages (Fig. 4C). Thus, extracellular DnaK has immune modulatory effects in both bone marrow-derived and peritoneal macrophages.

### DnaK-treated macrophages promote melanoma growth in mice

To test whether the M2-like macrophages generated by DnaK treatment were functional M2s, we tested their ability to promote tumor growth in an allogeneic murine melanoma model. We co-injected BMMs previously treated with DnaK or M1 macrophages, or untreated BMMs, with B16F10 (B16) cells (I-A^b^) in BALB/c mice (I-A^d^) and followed tumor growth over several days.

**Figure 5:**
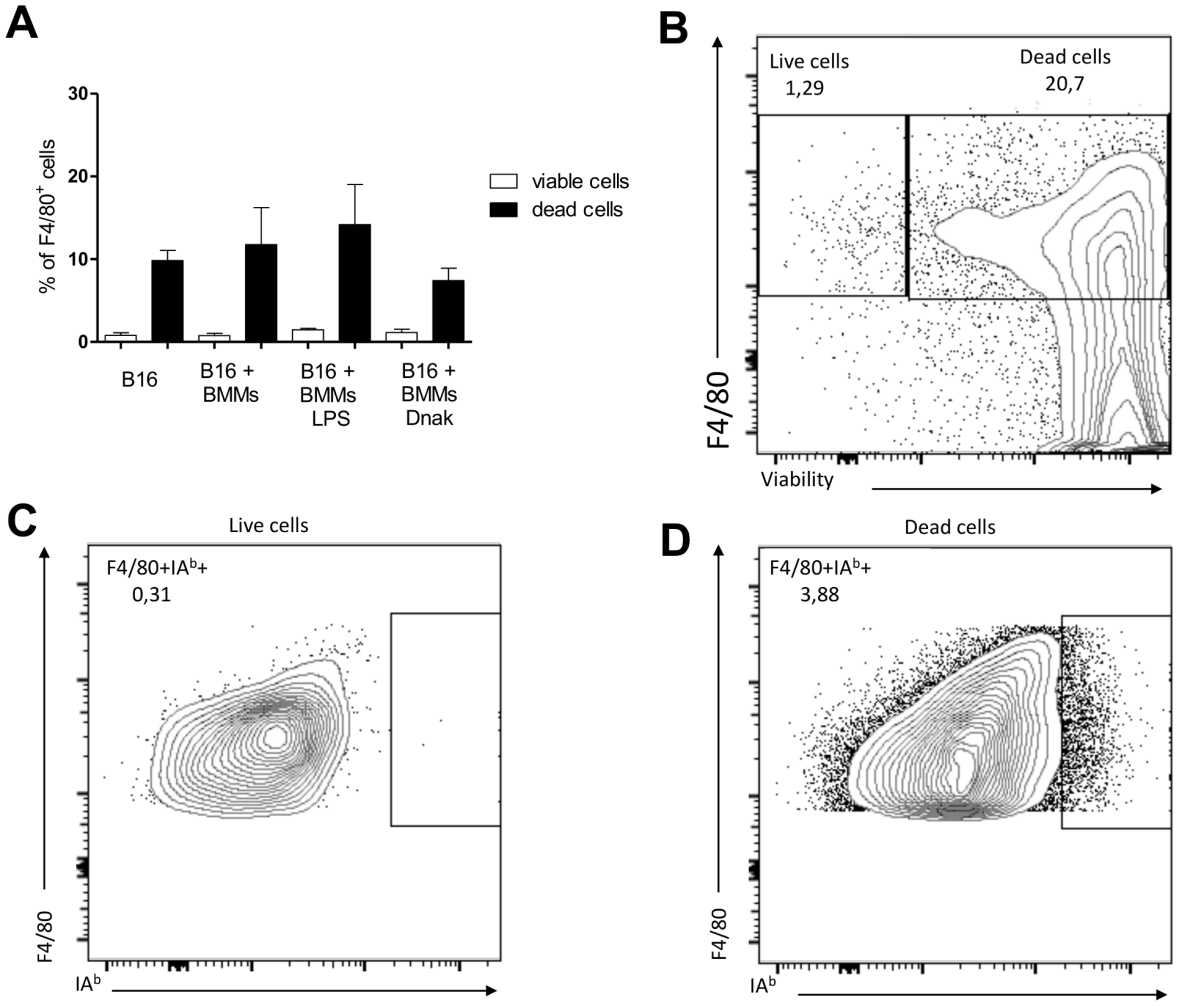
Viability of tumor infiltrating F4/80^+^I-A^b+^ cells 16 days after co-injection. All mice were euthanized and tumors dissected and digested with collagenase D. The single cell suspension obtained was stained for flow cytometry, with antibodies against MHC class II allotype (I-A^b+^), F4/80 as well as viability stain. (A) Bar graph with the percentages of tumor infiltrating F4/80^+^ cells, dead or alive; (B) Dot plot representative of the difference in viability of these two populations; (C) representation of F4/80^+^IA^b+^ in the viable population; (D) representation of F4/80^+^I-A^b+^ in the non-viable population.

On the 16th day after co-injection of B16 tumors and BMMs, the mice were euthanized and their tumors removed and dissected. Tumors were first digested with collagenase D and the cells in a single cell suspension were stained with antibodies for flow cytometry analysis. The results of this analysis are shown in Figure 5. Sixteen days after co-injection of tumors and treated allogeneic BMMs, these macrophages can no longer be found alive inside the tumors. In fact, most of the macrophages inside the tumors are not viable (Figure 5, A and B). Of the viable macrophages found infiltrating the tumors, none are IA^b^+ (Figure 5C).

**Figure 6:**
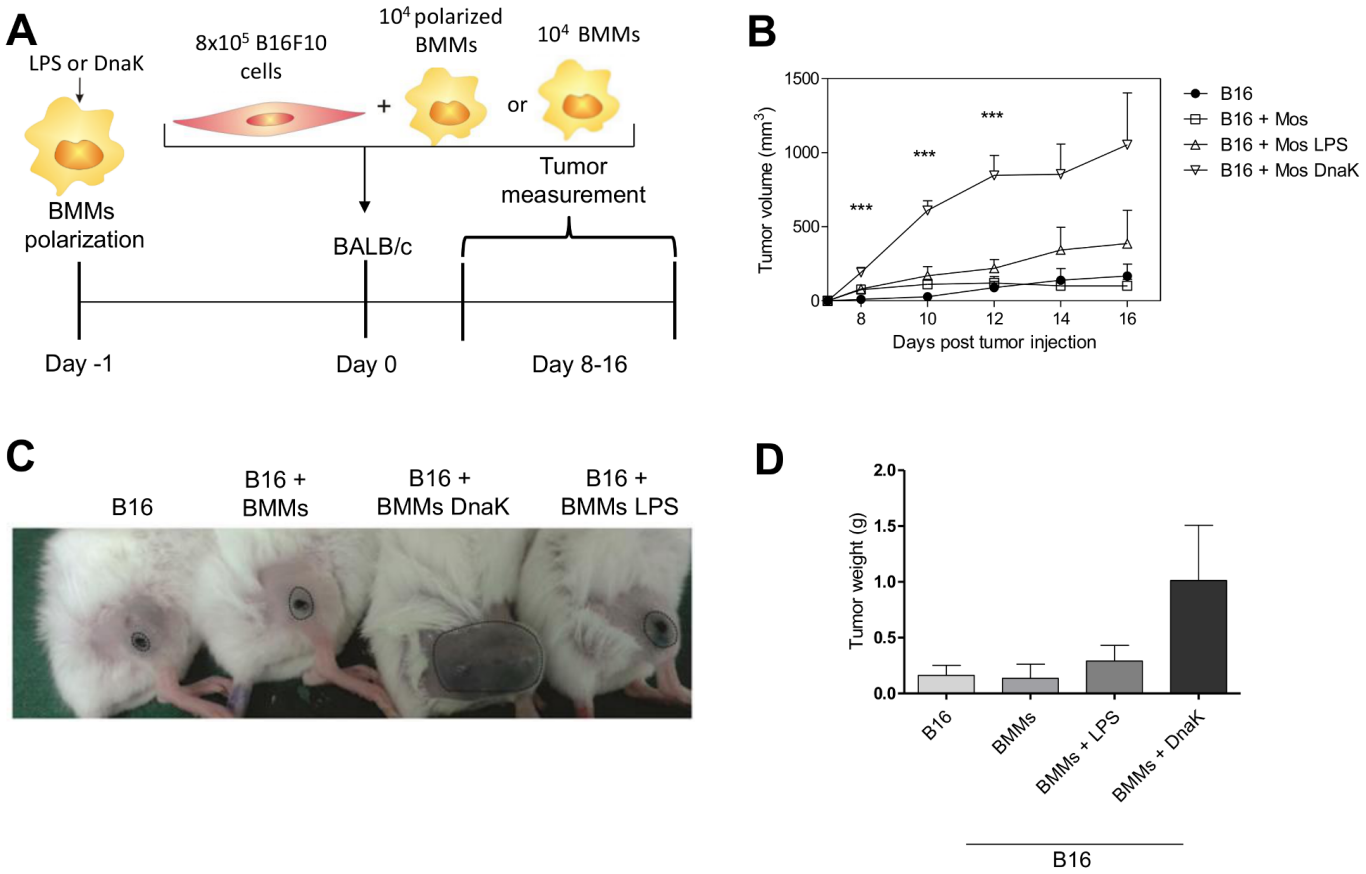
DnaK-treated macrophages enhance tumor growth in murine allogeneic melanoma model. (A) The murine melanoma cell line B16F10 was co-injected with macrophages exposed to 30 µg/mL DnaK, 30 ng/ml of LPS, untreated macrophages, or no other cells for 24 h as illustrated in experimental design. (B) Cells were subcutaneously injected into BALB/c mice (4 mice per group) and the tumor volume was measured 8 days later as indicated. The values represent means ± SEM. (*) p<0.05 and (***) p<0.001 indicate significant difference between the macrophages exposed to DnaK in relation to B16 group. The data were analyzed by one-way ANOVA with Tuckey post hoc test in each time point. (C) Macroscopically view of tumor size. (D) Tumor weight on day 16 after tumor injection. The values represent means ± SEM. All data representative of three independent experiments.

Figure 6 shows the results for tumor growth upon injection with polarized BMMs. Tumors co-injected with M1 macrophages, untreated macrophages, or alone could not develop in BALB/c hosts (Fig. 6). Nevertheless, when B16F10 cells were injected together with DnaK-polarized macrophages, tumors were capable of growth in the allogeneic host (Fig. 6B). This difference in tumor growth could be observed macroscopically (Fig. 6C). In addition, tumors that were co-injected with DnaK-treated macrophages were bigger when compared to other groups (Fig. 6D). Altogether, these findings showed that extracellular DnaK induces M2-like macrophages with tumor-promoting potential.

## Discussion

In this study we provide functional evidence that DnaK from *Mycobacterium tuberculosis* skews macrophages towards the M2 phenotype. This may, at least in part, explain the role of DnaK in mycobacterial virulence.

Some of the strategies used by mycobacteria to polarize macrophages to M2 have been described. *M. tuberculosis* can secrete lipoarabinomannan which inhibits IFN-γ induced macrophage activation [8]. In addition, mycobacteria can shift macrophages to M2 by inducing IL-10 [28], [29]. Indeed, high levels of macrophage-derived IL-10 are correlated with active TB in humans [30], [31]. After efficient treatment of TB patients, this anti-inflammatory cytokine profile shifts to inflammatory [31], [32]. Also, in human macrophages infected with *M. tuberculosis*, the repression of IL-10 leads to an enhancement of phagosome protease activity, leading to a higher eradication of the pathogen [33].

*In vivo*, macrophage polarization induced by IL-10 is associated with the production of this cytokine in infected organs [30], [34] and tumor microenvironment [35], [36]. To assess the role of IL-10 specifically produced by macrophages, Schreiber *et al.* infected macrophages from mice in which IL-10 was upregulated in these cells with *Mycobacterium tuberculosis* [28]. These animals were more susceptible to infection, died early and exhibited a higher bacterial load in the lungs. In the same study, *M. tuberculosis* infected macrophages had a M2 phenotype. In *Toxoplasma gondii* infected RAW 264.7 macrophages, the parasite Hsp70 inhibited production of NO and NF-kB activation, resulting in increased parasite load [37].

In both cases, macrophage modulation is a major strategy to evade effector immune responses, avoiding tumor destruction and pathogen eradication. We show that DnaK induce the production of IL-10 by macrophages and polarization to M2 phenotype. Because most of the co-injected allogeneic macrophages are found dead inside the tumors on day 16, we believe that the effect of the DnaK polarized BMMs is very robust and occurs very early, allowing the implantation of the tumor in the host. It is possible that DnaK acts as an immunomodulator with a putative virulence role in bacterial infections, polarizing macrophages to M2, with production of IL-10.

DnaK was found within vesicle membranes released by mycobacteria which can modulate macrophages in a pathway dependent on TLR2 [26]. Other molecules from *Mycobacterium tuberculosis*, like the PPE18 protein, induce IL-10 production through TLR2 in order to evade effector immune responses mediated by CD4+ T cells [38], [39]. Chalmin et al. demonstrated that both murine and human HSP70 present in exossome membranes released from tumor cells enhances immunosuppressive functions of MDSCs [40], leading to tumor growth, in a TLR-2, IL-10 dependent mechanism. It is thus possible to hypothesize that other members of the Hsp70 family, when released from infected or tumor cells in vesicles, can engage a TLR2 pathway, leading to IL-10 production in myeloid cells, and polarizing macrophages to an M2 phenotype. This pathway would be activated both in infections and tumors, and further experiments are necessary to test this hypothesis.

## Materials and Methods

### Mice

6 to 10-weeks-old female C57BL/6 and BALB/c mice were purchased from FEPPS (Rio Grande do Sul, BRA). All mice were housed in individual and standard mini-isolators (Techniplast) in an SPF facility (Institute of Biomedical Research – PUCRS) with free access to water and food. Mice used in experiments have a range of weight between 18–22 g. The method of euthanasia used was a carbon dioxide (CO2) chamber (Beiramar). All procedures were performed in accordance with the guidelines of the Federation of Brazilian Societies for Experimental Biology and approved by the Ethics Committee for the Use of Animals of Pontifícia Universidade Católica do Rio Grande do Sul (CEUA-PUCRS) under protocol ID CEUA 12/00316.

### Protein purification and LPS extraction

Recombinant DnaK of *Mycobacterium tuberculosis* was produced with the construct pET23a(+)/MtbDnaK in XL1-blue *Escherichia coli* and purified according to Mehlert [41]. To remove LPS, Triton X-114 was used according to the method described in Aida et al. [42]. Contaminating Triton X-114 was removed by incubating overnight with Bio-Beads (Bio-Rad) at 4°C with agitation, as described in [22]. Protein concentration was determined using Qubit Protein Assay Kit (Invitrogen) and the Qubit Fluorometer (Invitrogen).

### Macrophages cultures and polarization

Macrophages were derived from bone marrow of C57BL/6 WT mice. Cells (10^6^) were cultured in 24-well plates in serum-free medium AIM-V (Gibco) with 10 ng/mL of GM-CSF (Peprotech). At day 3, medium was collected and cells were cultured for a further 3 days in the presence of fresh AIM-V with 10 ng/mL of GM-CSF. On the seventh day of culture, the non-adherents cells were separated from adherent cells (macrophages) and stimulated as described below. The purity in BMMs cultures was higher than 90% as assessed by staining with anti-F4/80 antibodies (data not shown).

Peritoneal macrophages were collected by peritoneal cavity wash with 5 mL of sterile serum-free AIM-V medium (Gibco). The cells were washed twice with sterile PBS and suspended in AIM-V, transferred to a 24 multi-well plates and allowed to attach for 30 min. Unattached cells were washed out with medium. The adherent cells, mainly peritoneal macrophages, were used for the experiments thereafter. Macrophages were evaluated by microscopic examination with May-Grunwald and Giemsa stains, indicating macrophage purity higher than 80%. Purity was confirmed by flow cytometry, using the F4/80 Ab (data not shown).

The obtained macrophages (from bone marrow or peritoneum) were stimulated for 24 h in serum-free AIM-V with 30 or 60 µg/mL of DnaK or left unstimulated. For the generation of classically or alternatively activated macrophages, cells were stimulated with LPS (30 ng/mL) or IL-4 (40 ng/mL) (both purchased from Peprotech) for 24 h, respectively.

### Arginase Assay

Arginase activity in cell lysates was measured based on the conversion of L-arginine to L-ornithine and urea according to the technique described by Corraliza and collaborators [43] with minor modifications. Briefly, cells were lysed for 30 min with 40 µL of PBS containing 0,1% Triton-X-100. 30 µL of 25 mMTris-HCl, pH 7.4 and 10 µL of 10 mM MnCl_2_ were added and the enzyme was heat-activated for 10 min at 56°C. Similar amounts of samples (40 µL) and 0.5 M L arginine (pH 9.7) were mixed and incubated for 1 h at 37°C. The reaction was stopped by adding 400 µL of H_2_SO_4_ (96%), H_3_PO_4_ (85%), H_2_O (1/3/7, v/v/v). The urea concentration was measured at 540 nm after the addition of 8 µL of α-isonitropropiophenone 6%, followed by heating at 95°C for 30 min. Values were compared with a standard curve of urea concentration.

### Nitrite Assay

Nitrite concentrations were measured using the Greiss reaction [44]. We used the Greiss Reagent Kit for Nitrite Determination (Molecular Probes), according to manufacturer's instructions. Samples were quantified by spectrophotometry at 540 nm using sodium nitrite as standard.

### Flow cytometry

The Fc receptors of macrophages were blocked with 24G2 supernatant containing 10% mouse serum and 10% rat serum, and later stained for F4/80 (BM8) from eBioscience; CD80 (16-10A1) from BD Biosciences; and CD206 (MR5D3) from AbDSerotec. Cells were analyzed using FACSCanto II (BD Biosciences) and BD FACSDiva software (BD Biosciences). Data obtained were analyzed using Flowjo software (version 7.6.5, Tree Star).

On Day 16 after the subcutaneous co-injection of polarized BMMs and B16 tumor cells, the mice were euthanized and tumors were excised and digested with collagenase D (Roche). The single cell suspension obtained was filtered to eliminate debri and stained for flow cytometry, using antibodies to F4/80, IA^b^, CD86 and viability (Fixable Viability Dye from eBioscience).

### Total RNA isolation and cDNA synthesis

Total RNA was isolated from murine macrophage cultures using RNAeasy kit (Qiagen) according to manufacturer's instructions. The concentration of the purified total RNA samples was measured using a Qubit RNA Assay Kit (Invitrogen) and the Qubit Fluorometer (Invitrogen). We added 50 ng of RNA each cDNA synthesis reaction using the SuperScript-III RT pre-amplification system (Invitrogen, Carlsbad, CA, USA). cDNA concentrations were measured using Qubit dsDNA HS Assay Kit (Invitrogen) and the Qubit Fluorometer (Invitrogen).

### Real time PCR

Real time PCR was carried out StepOne Real-Time PCR System (Applied Biosystems) using Platinum SYBR Green qPCRSuperMix-UDG (Invitrogen) following the manufacturer's instructions. The thermal cycling conditions included an initial denaturation for 2 min at 95°C and 40 cycles consisting of a denaturation step at 95°C for 15 s, an annealing step at 60°C for 30 s and an extension step for 1 min at 70°C. Samples was analyzed in triplicates. The relative mRNA levels were calculated using the comparative Ct method [45], using the house keeping gene β-microglobulin as a normalizer. Non-treated macrophages served as a reference for treated macrophages.

Primers sequences we used for β-microglobulin were F: TCCTGGCTCACACTGAATTC and R: CTGCGTGCATAAATTGTATAGCA; for Fizz1 (Retnla) F: TCCCAGTGAATACTGATGAGA and R: CACTCTGGATCTCCCAAGA; and for Ym1 (Chi3l3) F: GGGCATACCTTTATCCTGAG and R: CCACTGAAGTCATCCATGTC

### Cytokines release measurement

Supernatants of cell cultures were analyzed for the presence of TNF, IL-10, MCP-1, IL-6, IL-12p70 and IFN-γ with the CBA Mouse Inflammation kit (BD Biosciences), according to manufacturer's instructions. Samples were analyzed using FACSCanto II (BD Biosciences) and BD FACSDiva software (BD Biosciences). Data obtained were analyzed using FCAP Array software (version 3.0, Soft Flow, Inc.) and expressed in pg/ml. TGF-β measurements were made using a human/mouse TGF-β1 (2nd Gen) ELISA Ready-SET-Go! kit (eBioscience), according to the manufacturer's instructions.

### Tumor and BMMs co-injection

The murine melanoma cell line B16F10 (ATTC CRL-6475) was cultured with DMEM media (Cultilab) supplemented with 10% of fetal calf serum (FCS) (Cultilab), 1× essentials amino acids (Gibco), 1× vitamins (Gibco) and 55 µM of β-mercaptoethanol at 37°C with 5% of CO_2_ atmosphere.

B16F10 cells (8×10^5^) were co-injected with 10^4^ of BMMs treated with LPS or DnaK as previously described in 100 µL of serum-free RPMI. Injections were performed subcutaneously in the thigh of male BALB/c or C57BL/6 mice, after anesthesia with 83 mg/kg of ketamine and 17 mg/kg of xylazine. Mice were photographed and tumor growth was evaluated using a digital caliper (Mitutoyo) in days 8, 10, 12, 14 and 16 post tumor injections. We used a modified ellipsoid formula 0.52 (Length×Width^2^) to access the tumor volume [46]. On 16th day after tumor injection, mice were euthanized; the primary tumor was removed and weighted. All procedures were performed in the afternoon between 1–6 p.m. Tumor borders were drawn in photographs (Figure 5) using CorelDRAW (version 12.0).

### Statistical analysis

Statistical analysis was performed using the Prism software (version 5.00, Graphpad Software Inc.). The one-way ANOVA test was used to determine differences between groups. Multiple comparisons among levels were checked with Tukey post hoc test. Differences between specific points were determined by a t test. The level of significance was set at p<0.05.
